# Iron Chelators and Antioxidants Regenerate Neuritic Tree and Nigrostriatal Fibers of MPP+/MPTP-Lesioned Dopaminergic Neurons

**DOI:** 10.1371/journal.pone.0144848

**Published:** 2015-12-14

**Authors:** Pabla Aguirre, Natalia P. Mena, Carlos M. Carrasco, Yorka Muñoz, Patricio Pérez-Henríquez, Rodrigo A. Morales, Bruce K. Cassels, Carolina Méndez-Gálvez, Olimpo García-Beltrán, Christian González-Billault, Marco T. Núñez

**Affiliations:** 1 Iron and Biology of Aging Laboratory, Biology Department, Faculty of Sciences, Universidad de Chile, Santiago, Chile; 2 Research Ring on Oxidative Stress in the Nervous System, Santiago, Chile; 3 Chemobiodynamics Laboratory, Chemistry Department, Faculty of Sciences, Universidad de Chile, Santiago, Chile; 4 Facultad de Ciencias Naturales y Matemáticas, Universidad de Ibagué, Ibagué, Colombia; 5 Neuronal and Cellular Dynamics Laboratory, Biology Department, Faculty of Sciences, Universidad de Chile, Santiago, Chile; Florey Institute of Neuroscience and Mental Health, The University of Melbourne, AUSTRALIA

## Abstract

Neuronal death in Parkinson’s disease (PD) is often preceded by axodendritic tree retraction and loss of neuronal functionality. The presence of non-functional but live neurons opens therapeutic possibilities to recover functionality before clinical symptoms develop. Considering that iron accumulation and oxidative damage are conditions commonly found in PD, we tested the possible neuritogenic effects of iron chelators and antioxidant agents. We used three commercial chelators: DFO, deferiprone and 2.2’-dypyridyl, and three 8-hydroxyquinoline-based iron chelators: M30, 7MH and 7DH, and we evaluated their effects *in vitro* using a mesencephalic cell culture treated with the Parkinsonian toxin MPP+ and *in vivo* using the MPTP mouse model. All chelators tested promoted the emergence of new tyrosine hydroxylase (TH)-positive processes, increased axodendritic tree length and protected cells against lipoperoxidation. Chelator treatment resulted in the generation of processes containing the presynaptic marker synaptophysin. The antioxidants N-acetylcysteine and dymetylthiourea also enhanced axodendritic tree recovery *in vitro*, an indication that reducing oxidative tone fosters neuritogenesis in MPP+-damaged neurons. Oral administration to mice of the M30 chelator for 14 days after MPTP treatment resulted in increased TH- and GIRK2-positive nigra cells and nigrostriatal fibers. Our results support a role for oral iron chelators as good candidates for the early treatment of PD, at stages of the disease where there is axodendritic tree retraction without neuronal death.

## Introduction

A large body of evidence shows that disturbed iron homeostasis, often coupled to mitochondrial dysfunction, plays an important role in the development of common neurodegenerative diseases such as Alzheimer’s disease, Parkinson’s disease and Huntington’s disease [1–3]. Disturbed iron homeostasis also underlies a less typified group of disorders known as neurodegeneration with brain iron accumulation, which are characterized by the presence of high brain iron levels particularly within the basal ganglia [4, 5]. Iron accumulation occurs in the substantia nigra pars compacta (SNc) of various animal models of PD induced by neurotoxins, including 6-hydrodopamine [6], MPTP [7] and lactacystin [8].

The persistence of a high iron phenotype in damaged areas, in conjunction with the known capacity of iron to generate harmful reactive oxygen species (ROS), provides the basis of the “metal-based neurodegeneration” hypothesis. According to this hypothesis, redox-active metals like iron generate ROS that cause peroxidation of membrane phospholipids, leading in turn to the formation of reactive aldehydes, which react with proteins producing misfolded aggregates that overwhelm the ubiquitin/proteasome protein degradation system and accumulate within intracellular inclusion bodies [9].

Iron chelation has been introduced as a novel therapy concept for the treatment of PD and other diseases with an iron accumulation component, as detailed in recent reviews [10–12]. Two 8-OH quinoline-based chelators that permeate the blood-brain barrier, M30 and PBT2, are regarded as putative therapeutic agents for the treatment of neurodegenerative diseases with an iron accumulation component [13, 14]. The 8-OH quinoline-based chelator PBT2 was used in a double-blind, randomized, placebo-controlled Phase II trials for Alzheimer’s disease. Patients in the group treated with 250 mg PBT2 daily showed significant improvement on a neuro-psychological test battery within 12 weeks of treatment [15]. Importantly, in a recent placebo-controlled randomized clinical trial, early-stage Parkinson's patients treated with deferiprone (DFP; 30 mg/Kg body weight) showed significantly decreased iron deposits in *substantia nigra* and significantly improved Unified Parkinson’s Disease Rating Scale motor indicators of disease progression [16]. The authors concluded that these results warrant a comprehensive evaluation of iron chelation therapy in PD.

Central nervous system neurons have axodendritic trees that contain thousands of excitatory and inhibitory synapses [17, 18]. Retraction of the axodendritic tree, a process called “dying-back,” results in neuronal dysfunction, which precedes neuronal death and the subsequent appearance of clinical symptoms [19–21]. Indeed, studies of post-mortem tissue from PD patients or from mice injected with 6-hydroxydopamine show significantly decreased axon length and dendritic spine density in neurons of the prefrontal cortex, the putamen and the caudate nucleus [22–25]. We recently reported that inhibition of mitochondrial complex I by sub-lethal concentrations of MPP+ results in dramatic shortening of the axodendritic tree of mesencephalic dopaminergic neurons without death of the neuronal soma [26]. Co-incubation of MPP+ with antioxidants or the use of low-iron medium prevents this axodendritic tree shortening, an indication that iron-induced oxidative damage mediates neurite retraction [26].

In the present work, we studied in mesencephalic cultures the effects of various iron chelators and antioxidant agents on axodendritic tree regeneration, previously collapsed by MPP+ treatment, and investigated the effects of the iron chelator M30 on the restoration of nigrostriatal fibers in MPTP-treated mice.

## Materials and Methods

### Animals

Two-and-a-half-month-old male C57Bl/6 mice and 14-day pregnant Sprague-Dawley rats were obtained from the Institute of Public Health, Chile. Mice were housed with a 12 h light, 12 h dark cycle. This study was carried out in strict accordance with the recommendations of the Assessor Committee in Bioethics guidelines from the National Fund for Scientific and Technological Development (FONDECYT, Chile). The protocol was approved by the Ethics Committee of the Faculty of Sciences, Universidad de Chile. All surgery was performed under sodium pentobarbital anesthesia, and all efforts were made to minimize animal suffering. Animals were monitored once a day during the overall length of the experiment. Examination included general aspect, possible loss of body weight, spontaneous and behavior upon prodding. No animal died as a result of the treatment with MPTP and later with M30. Because of male-male aggressive behavior, occasionally there were individual aggressions that resulted in injuries. In these cases, the injured animal was terminated according to approved protocols. Mildly affected animals were given the analgesic ketoprofen (2–5 mg/Kg body weight). Typically 3 victims of aggression died in a cohort of 24 animals. The protocol used to determine euthanization was based in a punctuation of the following observations: 1) General aspect: normal 0; uneven fur: 1; ocular or nasal secretions: 2; abnormal posture: 3. 2) Body weight loss: none: 0; less than 10%: 1; between 10 and 20%: 2; over 20%: 3. 3) Spontaneous behavior: normal: 0; small changes: 1; inactivity: 2; very unquiet on no movement: 3. 4) behavior upon prodding: normal: 0; small changes: 1; moderate changes: 2; aggressive or comatose animals: 3. Mice were euthanized when having an accumulate score of 10–12 points. Animals were euthanized by an i.p. injection of sodium pentobarbital.

### Reagents

1-methyl-4-phenylpyridinium (MPP+), 1-methyl-4-phenyl-1,2,3,6-tetrahydropyridine (MPTP), desferrioxamine (DFO), deferiprone (DFP) and 2,2’-dipyridyl (DPD), M30, N-acetylcysteine (NAC) and dymetylthiourea (DMTU) were from Sigma-Aldrich (St. Louis, MO).

The structures and synthetic strategies for 7-morpholinylmethyl-8-hydroxyquinoline (7MH) and 7-dimethylaminomethyl-8-hydroxyquinoline (7DH) are described in S1 Fig.

### Mesencephalic cell culture

Mesencephalic cells were prepared as described [27]. On day 14 of gestation, pregnant Sprague-Dawley rats were exposed to CO_2_ followed by laparotomy. The fetuses were collected in cold L-15 medium and the brains were isolated. The mesencephalic dopaminergic region (A8, A9 and A10 dopaminergic nuclei) was dissected and dispersed by repeated pipetting in DMEM/F12 medium containing 0.1% bovine albumin, 5 mg/ml insulin, 30 nM L-thyroxin, 20 nM progesterone, 30 nM sodium selenite, 100 unit/ml penicillin, 100 mg/ml streptomycin and 5% fetal bovine serum. Cells were plated on glass cover slips pre-coated with 1 mg/ml poly-L-lysine, at a density of 55,000 viable cells/cm^2^. On the first day *in vitro* (DIV1), the medium was changed, and then half of the medium was changed at DIV3, DIV5 and DIV7. Cells were used at DIV7.

### Determination of the total length of the axodendritic tree

The total length of the axodendritic tree was determined using the HCA-Vision software (CSIRO, Australia). The total length of the axodendritic tree of DIV7 dopaminergic neurons ranged from 2400 to 2800 μm.

### Immunocytochemistry and immunohistochemistry

For immunocytochemistry, cells grown on cover slips were fixed in PBS containing 4% paraformaldehyde, 4% sucrose; after permeation with 0.2% Triton-X-100 in PBS, cultures were incubated overnight at 4°C with the primary antibody followed by 2 h incubation with the secondary antibody. The primary antibodies used in this study were rabbit anti-TH and goat anti-G-protein-regulated inward-rectifier potassium channel 2 (GIRK2) from Sigma-Aldrich, mouse anti-synaptophysin from EMD Millipore and mouse monoclonal anti- 4-hydroxynonenal antibody HNE-J2 from Abcam. The secondary antibodies (Invitrogen) were anti-mouse IgG labeled with Alexa Fluor 488 or 546, anti-rabbit IgG labeled with Alexa Fluor 488 or 546 and anti-goat IgG labeled with Alexa Fluor 633. TOPRO-3 staining (Invitrogen) was used for nuclei identification. Cells were viewed with a Zeiss LSM Meta confocal laser scanning microscope (Carl Zeiss, Göttingen, Germany).

Immunohistochemistry was performed as described [28]. Briefly, mice brains were fixed by transcardiac perfusion of 4% paraformaldehyde in PBS, dissected and post-fixed for 24 h in the same fixation solution. Parasagittal slices of 75 μm width, with a 10-degree tilt, were obtained in a cryostat as described [29]. This technique, named the “Basal Ganglia Slice” technique, allows for sequential cuts along the nigrostriatal pathway. Free-floating slices were permeabilized, blocked for nonspecific binding sites and incubated with primary antibody. Immunolabeling was visualized using Alexa 488- or Alexa-546-tagged secondary antibodies. For detection of the nigrostriatal pathway, the slice with the highest TH-labeling intensity was selected as representative.

### Axodendritic tree recovery protocol

**Fig 1:**
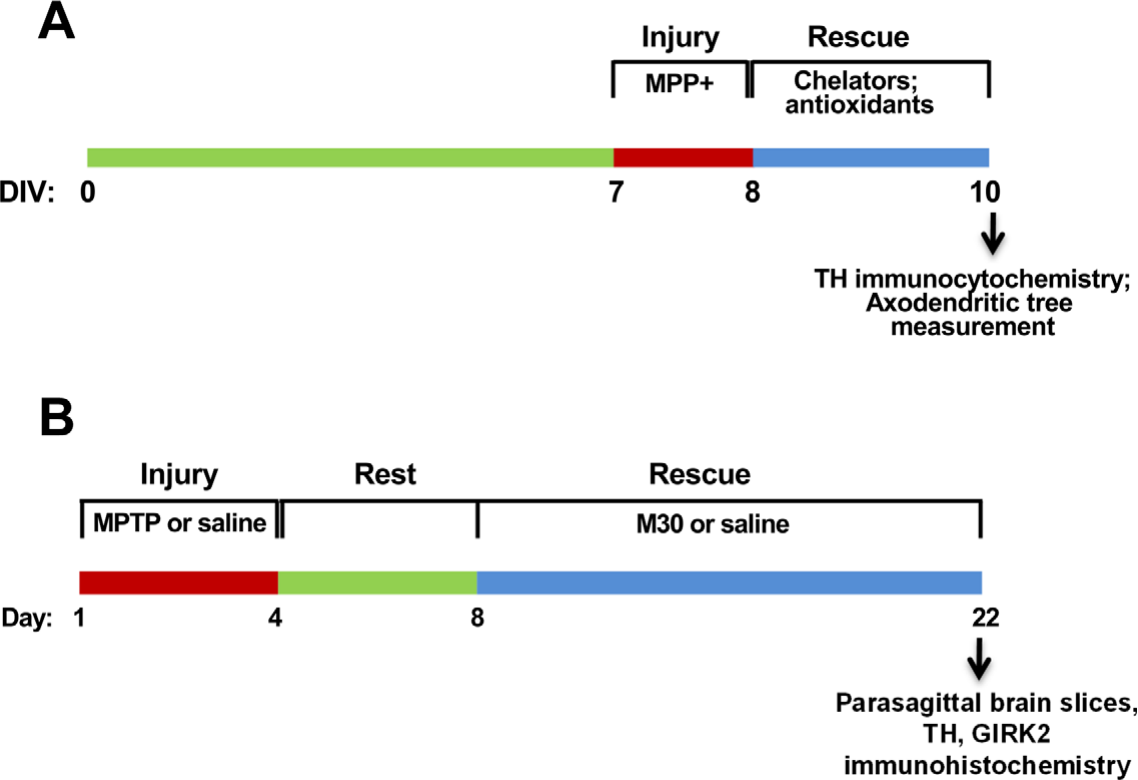
Injury and regeneration protocols. A) *In vitro* axodendritic tree regeneration. Mesencephalic cells at DIV7 were treated for 24 h with 0.5 μM MPP+, followed by treatment for 48 h with chelators or antioxidants. Control cells were incubated for 72-h in regular culture medium. B) *In vivo* recovery protocol. Mice (four per group) were treated for 4 days with four i.p. injections of either 25 mg/kg body weight of MPTP or saline. After a 4-day rest period, mice were given daily doses of either 2.5 mg/kg body weight of M30 or water for 14 days. One MPTP-treated group was sacrificed at day 8. The other four groups were sacrificed at day 22.

Mesencephalic cells at DIV7 were treated with 0.5 μM MPP+ for 24 h in complete culture medium. No indication of TH-positive neurons death was evident as determined by the ratio of TH-positive neurons to overall cells, specified by TOPRO3 staining [26]. MPP+ was removed and cells were subsequently incubated for 48 h in complete medium with or without (Control) an iron chelator at the following concentrations: DFO: 5 μM; DFP: 50 μM; DPD: 10 μM; 7MH: 50 nM; 7DH: 50 nM and M30: 0.25 μM. The concentrations used for the different chelators were determined as optimal on the basis of experiments of dose of chelator vs. axodendritic tree length in the absence of MPP+ treatment (chelator toxicity test) and dose of chelator vs. axodendritic tree recovery after MPP+ treatment (recovery activity test). NAC was used at either 0.5 or 1 mM and DMTU at 1 or 2 mM. A scheme of the axodendritic tree recovery protocol is shown in Fig 1A.

### Recovery of nigrostriatal fibers protocol

We used a modification of the protocol described by Gal et al. [30], schematically shown in Fig 1B. In brief, C57BL/6 mice were subjected to sub-acute MPTP intoxication, in which mice were injected i.p. once a day for four consecutive days with either 25 mg/kg body weight of MPTP or with the same volume of saline. Mice were left to recover for four days. Subsequently, two saline-treated groups and two MPTP-treated groups were dosed by gavage once a day for 14 consecutive days either with 2.5 mg/kg of body weight of M30 or with the same volume of saline. All the experiments were performed with three mice per experimental group. For quantification of TH labeling, the sum of all the pixels in fixed areas encompassing either SNc or nigrostriatal fibers was determined using the Quantity One software program (Bio-Rad Inc). This protocol was applied twice with similar results.

### Statistical analysis

The Shapiro-Wilk test was used for the determination of normal distribution of replicates. One-way ANOVA was used to test for differences in mean values, and Tukey’s post-hoc test was used for comparisons between mean values. A value of P<0.05 was taken as statistically significant.

## Results

**Fig 2:**
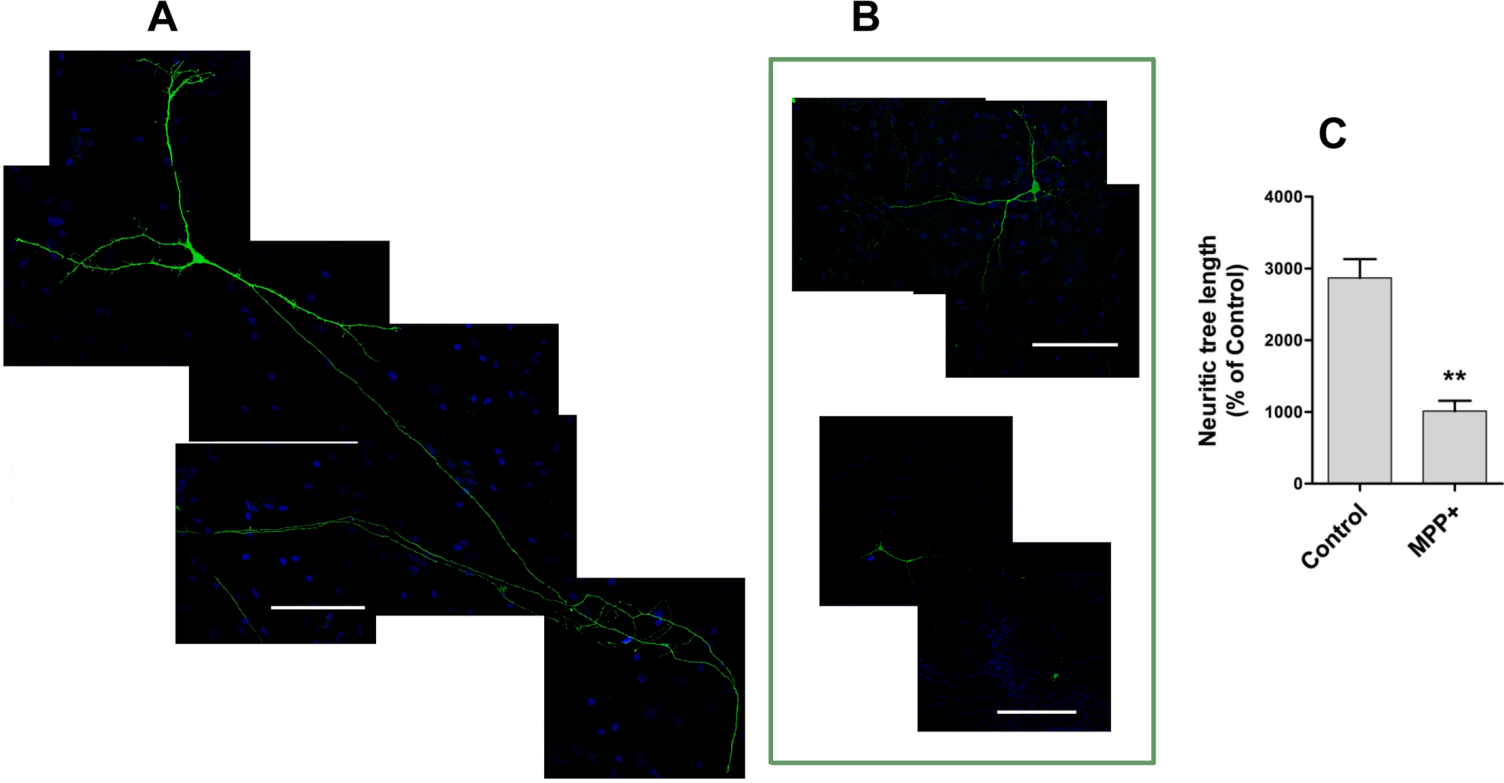
Total neuritic tree length of control and MPP+-treated TH-positive neurons. Mesencephalic cells (DIV7) were treated for 24 h with 0.5 μM MPP+, after which dopaminergic neurons were identified by TH immunostaining; the cell nucleus was stained with TOPRO (blue). A) Seven-frame composition of a representative control neuron. B) Images of two representative MPP+-treated neurons. C) Quantification of total neuritic tree length determined with the HCA Vision program. Values are Mean ± SEM for Control (N = 20 neurons) and MPP+-treated cells (N = 11 neurons).

In order to study putative axodendritic tree regeneration, we first analyzed whether sub-lethal doses of MPP+ promote axodendritic tree shortening in dopaminergic neurons without causing cell death. As illustrated in Fig 2, we found that treatment for 24 h with 0.5 μM MPP+ markedly decreased neuritic tree length. Fig 2A shows a control TH-positive neuron, with a long axon and numerous neurites, and Fig 2B shows two neurons subjected to incubation for 24 h with 0.5 μM MPP+, which produced a 65% decrease in neuritic tree length relative to the control (Fig 2C). Under the assay conditions used (24 h, 0.5 μM MPP+), no cell death was observed. Evaluation of TH-positive cell versus total (TOPRO-positive) cells indicated a ratio of 0.0072 ± 0.0012 for control cultures and 0.0067 ± 0.0010 for MPP+-treated cultures.

**Fig 3:**
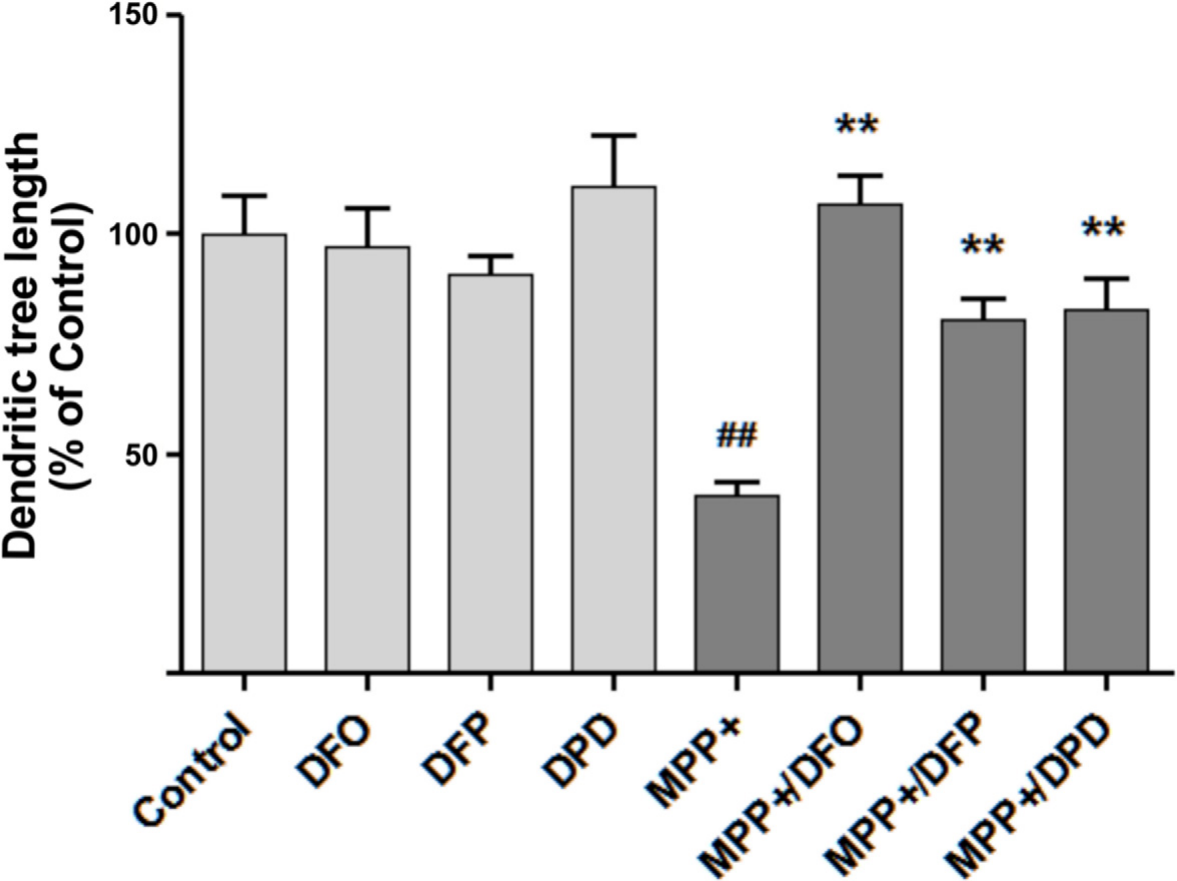
Iron chelators restore the axodendritic tree in MPP+-treated DA neurons. Mesencephalic cells in culture were treated as described in the legend to Fig 1A for 24 h with 0.5 μM MPP+ followed by treatment for 48 h with 5 μM DFO, 50 μM DFP or 10 μM DPD. Cells were fixed and immunostained for TH. Total axodendritic tree length was determined as described in Methods. Values represent Mean ± SEM, N = 10 neurons for each experimental condition. ** P< 0.01 as compared to MPP+-treated cells. ^##^ P< 0.01 as compared to the Control condition.

Considering that the effect of MPP+ on neuritic tree length most probably depends on the intracellular iron concentration [26], it was of interest to investigate whether commonly used iron chelators could regenerate the axodendritic tree of cultured dopaminergic neurons previously damaged by MPP+. The chelators DFO, DFP and DPD were all effective in inducing neuritogenesis, restoring the axodendritic tree of MPP+-damaged neurons after 48 h of treatment (Fig 3).

The putative functionality of regenerated neurites was evaluated by analyzing the presence of the presynaptic marker synaptophysin, a synaptic vesicle protein that participates in neurotransmitter secretion [31]. Control TH+ neurons presented synaptophysin-positive spots in all new thin neurite processes; synaptophysin was also evident in neurites of cells treated with MPP+, and with MPP+ plus DFO, DFP or DPD (S2 Fig). Because TH-positive neurons represent less than 1% of the total cells in the culture, it was not possible to evaluate the changes in synaptophysin levels by quantitative methods like Western blot analysis.

**Fig 4:**
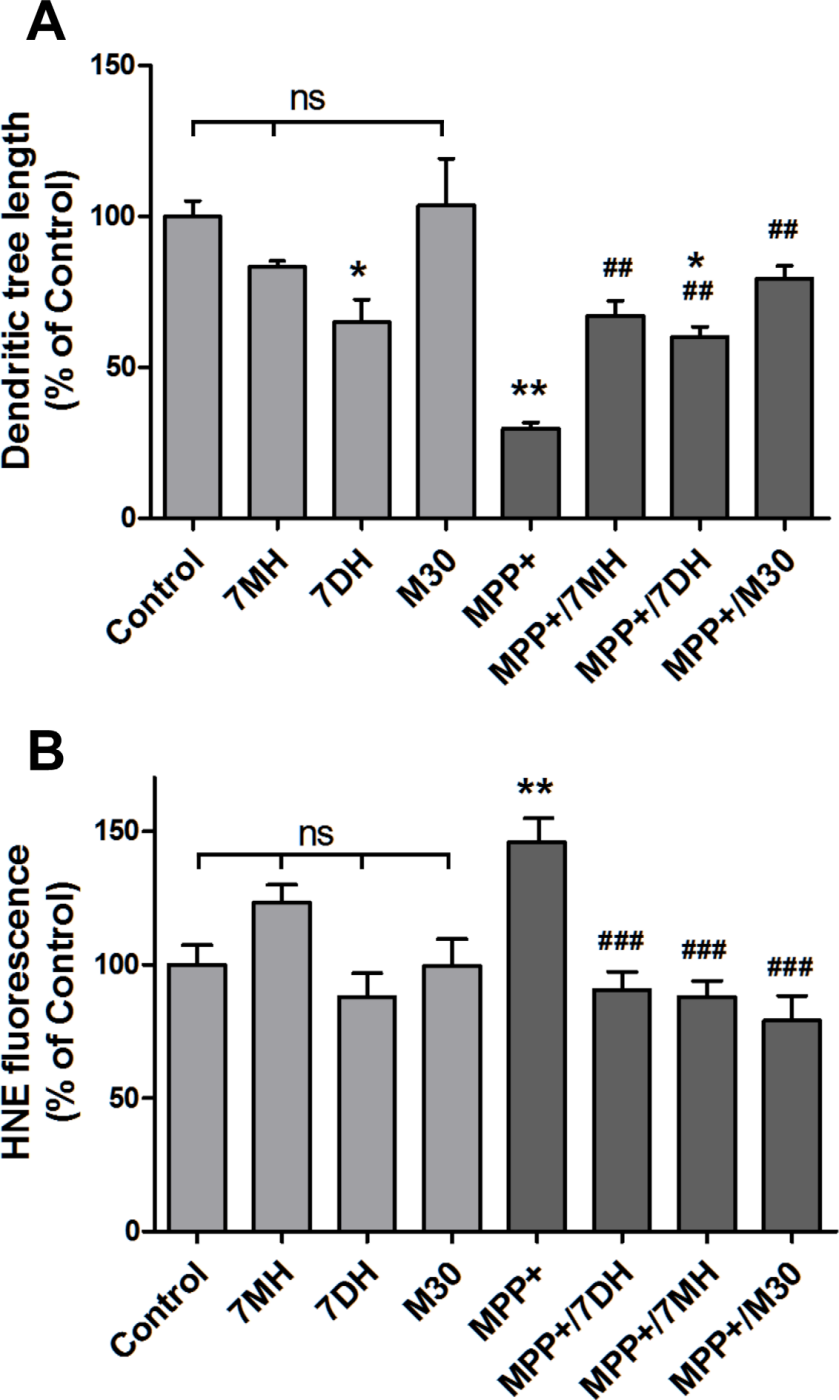
Effect of 8-hydroxyquinoline-based chelators on axodendritic tree restoration. A) Cells were treated for 24 h with 0.5 μM MPP+ and then for 48 h with 50 nM 7MH, 50 nM 7DH or 0.25 μM M30. The length of the axodendritic tree of TH-positive neurons was determined as described in Methods. Values represent Mean ± SEM of the axodendritic tree length determined in 10 neurons per experimental condition. * P< 0.05, ** P< 0.01 as compared to Control. ^##^ P< 0.01 as compared to MPP+-treated cells. B) Cells treated MPP+ and then with 7MH, 7DH or M30 as described in (A) were double immunostained with anti-TH anti-HNE. HNE immunostaining intensity was determined in TH-positive neurons with the ImageJ program. Values normalized to Control. ** P< 0.01 as compared to Control; ^###^ P< 0.001 as compared to MPP+-treated cells; ns, non-significant.

We tested next the regenerative capacity of three 8-hydroxyquinoline-based iron chelators. Treatment with M30 resulted in significant regeneration of the axodendritic tree of MPP+-damaged neurons (MPP+ vs MPP+/M30, P< 0.01) (Fig 4). Significant regeneration activity was observed also upon treatment with 7MH (MPP+ vs MPP+/7MH, P< 0.01) and 7DH (MPP+ vs MPP+/7DH P< 0.01), whereas treatment with 50 nM 7DH showed some toxic activity (Control vs /DH, P<0.05) (Fig 4A). Chelators also reduced lipoperoxidation, as determined by decreased levels of protein-HNE adducts (Fig 4B.

**Fig 5:**
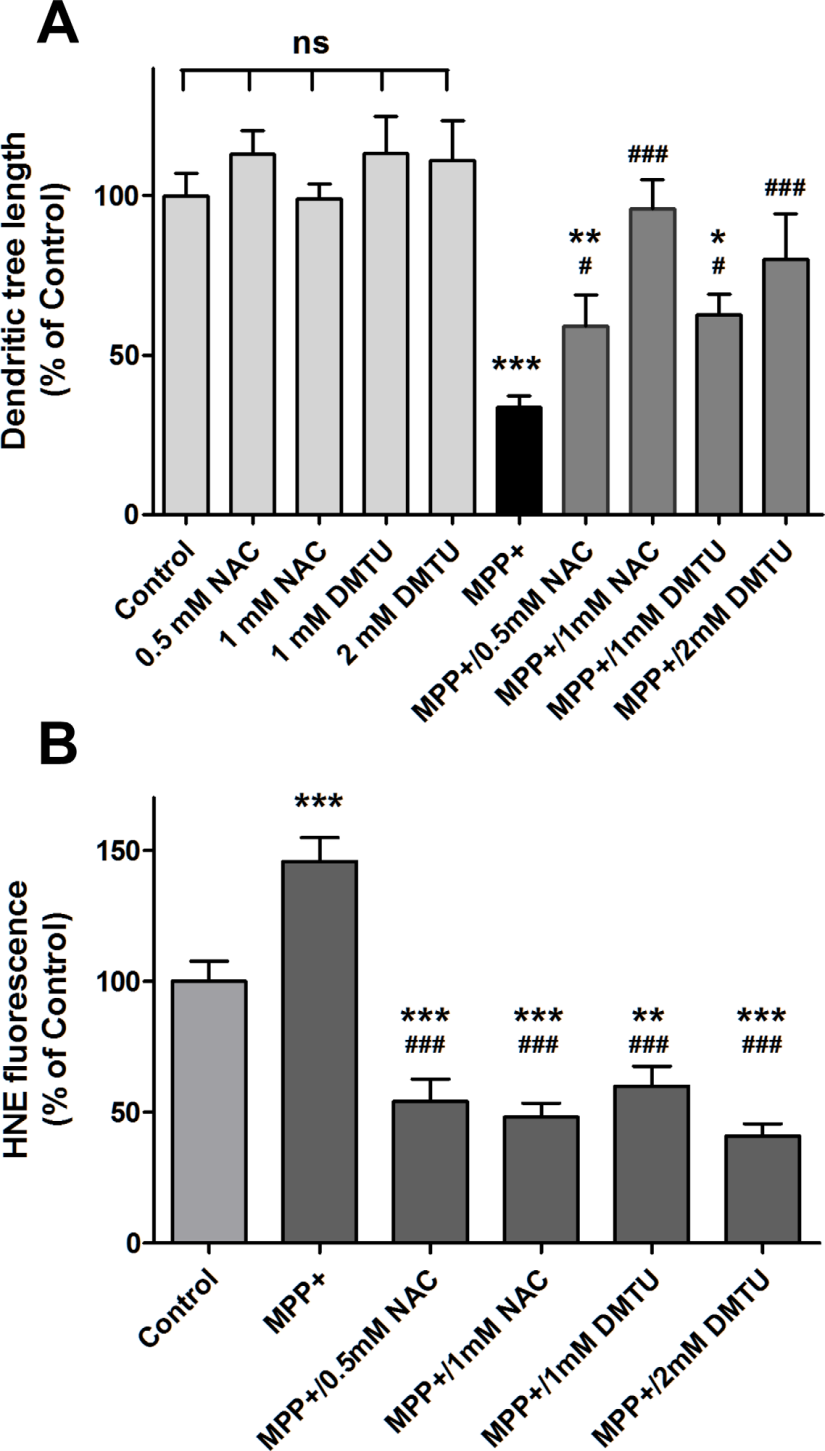
The antioxidants NAC and DMTU regenerate MPP+-damaged axodendritic tree. Mesencephalic cells at DIV7 were treated for 24 h with 0.5 μM MPP+, followed by treatment for 48 h with NAC (0.5 or 1 mM) or DMTU (1 or 2 mM). Control cells were incubated for 72 h in regular culture medium. Cells were immunostained for TH and total axodendritic tree length of TH+ cells was determined. Values represent Mean ± SEM. The number of neurons determined for each condition ranged between 12 and 28. *P< 0.05 compared to Control; **P< 0.01 compared to Control; ^#^P< 0.05 compared to the MPP+ condition; ^#^P< 0.05 compared to the MPP+ condition; ^##^P< 0.01 compared to the MPP+ condition; ^###^P< 0.001 compared to the MPP+ condition; ns, non-significant. B) Cells treated MPP+ and then with NAC or DMTU as described in (A) were double immunostained with anti-TH anti-HNE. HNE immunostaining intensity was determined in TH-positive neurons with the ImageJ program. Values normalized to Control. *** P< 0.001 as compared to Control; ^###^ P< 0.001 as compared to MPP+-treated cells.

Given the critical role played by ROS in PD neurodegeneration [32], we tested the possible mediation of reducing the cellular oxidative tone in the process of neuritogenesis. To this end, we examined the effects of the antioxidants NAC and DMTU on the recovery of the axodendritic tree after MPP+ treatment (Fig 5). Treatment for 48 h with 1 mM NAC or 2 mM DMTU resulted in almost complete recovery of the axodendritic tree of MPP+-damaged neurons, while treatment with 0.5 mM NAC or 1 mM DMTU afforded significant recoveries to 59% and 62% of control, respectively (Fig 5A). As expected, antioxidants also reduced MPP+-induced lipoperoxidation, as determined by decreased levels of protein-HNE adducts (Fig 5B).

**Fig 6:**
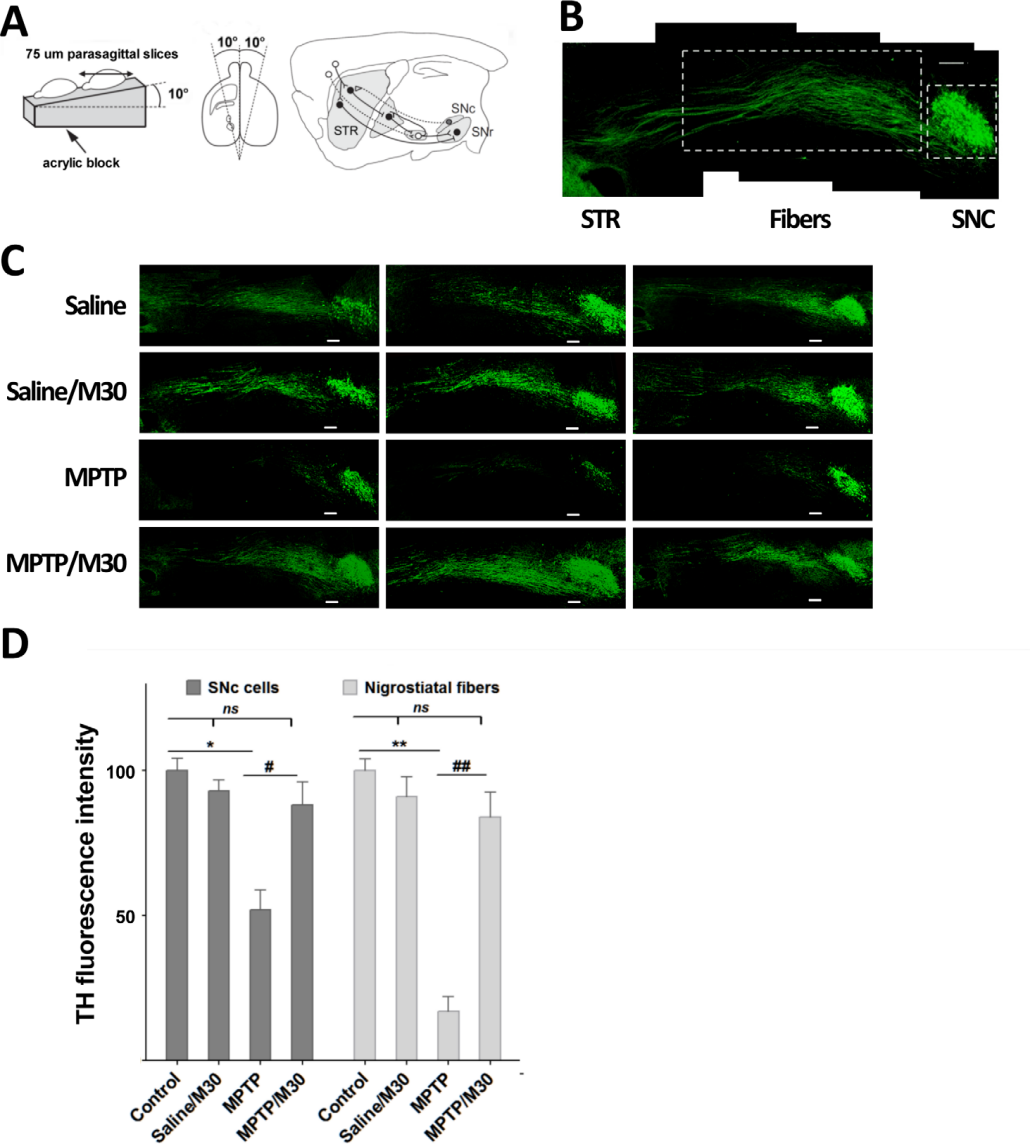
Recovery of SNc-striatum fibers by M30 treatment. A) Scheme of the “basal ganglia slice” procedure. Brains were placed on an acrylic base with a 10-degree slope in one of its faces, and were cut into 75 μm parasagittal slices. Image modified from [29]. B) Low-amplification photomontage of the nigrostriatal pathway. Shown is a five-frame reconstitution of the nigrostriatal pathway from a control animal. STR, striatum; SNc: substantia nigra pars compacta. Dashed frames highlight the regions used to quantify TH intensity in fibers and SNc (see below). C) Parasagittal slices. Shown are frames from individual mice subjected to the conditions described in Fig 1B, namely, Control Saline/Saline; Saline/M30; MPTP; MPTP/M30. Scale bar: 200 μm. D) TH labeling intensity of SNc and nigrostriatal fibers from frames of Fig 6C quantified with the Quantity One software program. Values represent Mean ± SEM of TH staining intensity normalized to Control, for either the SNc or the nigrostriatal fibers area. * P<0.05 and ** P< 0.01 compared to the control (saline/saline) condition. ^#^P<0.05 and ^##^ P< 0.01 compared to the MPTP condition.

Next we evaluated in an animal model of PD whether oral treatment with the 8-hydroxyquinoline-based chelator M30 could regenerate nigrostriatal fibers of neurons previously damaged by MPTP, using the protocol outlined in Fig 1B. The relative abundance of fibers and somas was determined by the intensity of TH immunolabeling. The nigrostriatal pathway was detected in 10-degree-tilted parasagittal sections obtained as described in Fig 6A. TH staining showed abundant fibers in the nigrostriatal pathway (Fig 6B). As compared to the Control (saline/saline) condition, treatment with MPTP reduced the intensity of TH labeling in the fibers and in the cell somas of the SNc (Fig 6C). Post-treatment with M30 (MPTP/M30) largely recovered this reduction. Quantification of the fluorescence intensity of the frames in Fig 6C revealed significant reductions in TH staining after MPTP treatment for both SNc neuronal cell bodies (52.1 ± 6.8%, P<0.05 compared to the Control condition) and fibers (17.3 ± 5.1, P< 0.01 compared to the Control condition) (Fig 6D). As compared with the MPTP condition, post-treatment with M30 (MPTP/M30) induced a significant increase of TH labeling in fibers (P< 0.01) and in SNc somas (P< 0.05).

**Fig 7:**
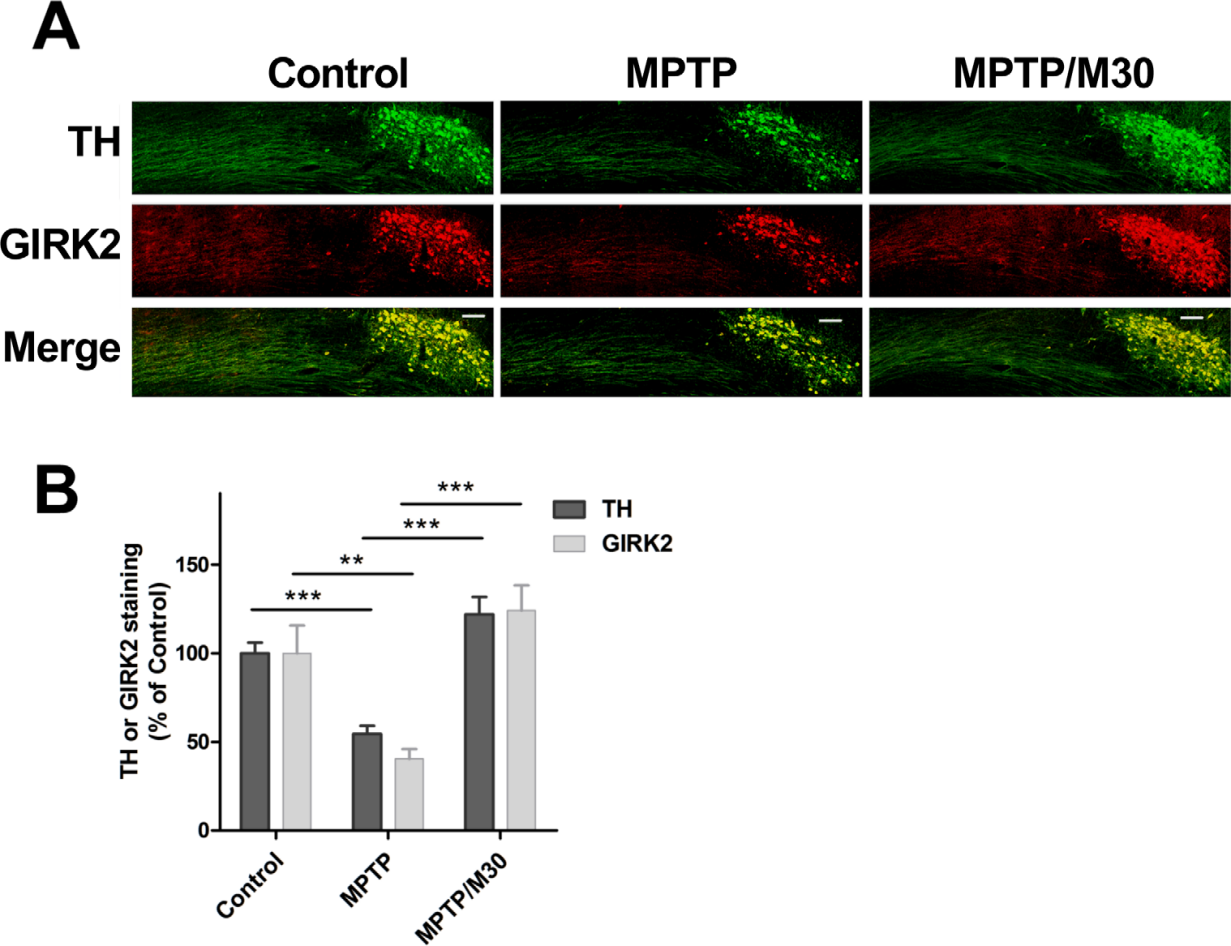
Recovery of GIRK2 immunostaining in SNc of MPTP-treated mice. **A)** Mice were treated for 4 days with daily injections of 25 mg/kg body weight of MPTP. The control group was injected with saline. After a 4-day rest period, MPTP-treated mice were split into 2 groups that were given by gavage 14 daily doses of either 2.5 mg/kg body weight M30 or saline (control). Parasagittal brain slices, obtained at day 22, were double immunostained for TH (green) and GIRK2 (red). Scale bar: 100 μm. Shown are representative images for the Control, MPTP and MPTP-M30 conditions. B) Quantification of TH and GIRK fluorescence in SNc soma. Data represent Mean ± SEM, N = 6 mice per experimental group. ** P<0.01; *** P< 0.001.

The above results suggest that M30 given after MPTP treatment results in the regeneration of nigrostriatal fibers and SNc cell somas. Nevertheless, it is possible that after MPTP treatment, fibers and cell somas were not lost but merely expressed decreased levels of TH [33–36]. Therefore, we analyzed a second axonal marker to test actual fiber and soma regeneration. The K^+^ channels GIRK1 and GIRK2 are important mediators of neuronal excitability *in vivo* [37]. In the central nervous system, GIRK2 is found in TH-positive neurons of the SNc and in a fraction of neurons of the ventral tegmental area [38]. We found that MPTP treatment diminished GIRK2 immunostaining in the nigrostriatal pathway and SNc neuronal somas, which recovered after M30 treatment (Fig 7A). GIRK immunofluorescence recovered to control levels after M30 treatment, in a way similar to TH immunofluorescence (Fig 7B). Neurons treated with M30 exhibited a tendency to increase in both TH and GIR2 immunofluorescence, but differences with controls did not reach significance. Because of low signal to noise ratio, no quantitative analysis of GIRK-positive nigrostriatal fibers was attempted.

## Discussion

Knowledge about factors that influence dopaminergic dendrite tree regeneration is incipient. Treatment with 9-methyl-beta-carboline afforded dendrite regeneration after rotenone or lipopolysaccharide intoxication [39]. Similarly, neurite growth was induced by immunophilin ligand GPI-1046 after 6-hydroxidopamine treatment [40]. In light of our previous report indicating that iron-generated ROS mediate the process of axodendritic tree collapse induced by MPP+ [26], here we studied whether iron chelators promote neurite regeneration both *in vitro*, using cell cultures of dopaminergic neurons treated with MPP+, and *in vivo* in an animal PD model of PD.

Incubation for a brief 48-h period with all six iron chelators tested (DFO, DFP, DPD, 7MH, 7DH and M30) effectively restored the axodendritic tree of MPP+-treated neurons, as determined by the emergence of new TH-positive processes. The recovered TH-positive neurites contained synaptophysin, a protein present in neurotransmitter-containing synaptic vesicles. These findings raise the possibility that treatment with iron chelators rescues functional axodendritic trees in MPP+-damaged dopaminergic neurons.

To test recovery of nigrostriatal fibers *in vivo*, we selected the iron chelator M30 in virtue of its permeability across the blood-brain barrier and its proven properties of neuroprotection and possible neurogenesis [30]. Treatment with M30 reduced iron content in the SN in two animal models of PD [8, 30]. In agreement with previous studies showing about 40% death of dopaminergic neurons under similar experimental conditions [41], we found that treatment with MPTP decreased the TH staining intensity of SNc neuronal cell bodies to 52% of control. Treatment with MPTP also resulted in a more pronounced loss of fibers in the nigrostriatal pathway to 17% of control. These results indicate that fibers are more susceptible than the cell body to MPTP-induced damage, and that in the process of neurodegeneration fiber loss precedes actual neuronal death, an observation consistent with a process of axon dieback. Oral supplementation with M30 for 14 days resulted in a striking recovery of TH staining, both in SNc cell bodies and nigrostriatal fibers. The recovery of SNc neuronal cell bodies and fibers as determined by TH staining was associated with recovery of the functional marker GIRK2, suggesting that the newly formed nigrostriatal fibers are functional.

The mechanisms underlying neurite regeneration by iron chelator treatment probably engage multiple factors. Neurite regrowth may result from the inhibition of HIF-prolyl hydroxylase [42–45], a Fe- and O_2_-dependent enzyme that mediates the degradation of transcription factor HIF-1α. Inhibition of prolyl hydroxylase by iron chelators may raise the level of HIF-1α and the expression of HIF-1α-dependent pro-survival genes, including *BDNF*, *GDNF* and *VEGF* [44]. Indeed, treatment with the chelators M30 or HLA20 induce neuritogenesis in mouse NSC-34 motor neuron cells, which is associated with the production of BDNF and GAP43 [46].

Changes in the oxidative tone may also contribute to neurite rescue. Iron chelators, by reducing the concentration of redox-active iron, may decrease also excessive ROS production in MPP+ treated neurons [47]. In fact, we found that antioxidants NAC and DMTU promoted the recovery of the axodendritic tree of MPP+-damaged neurons to a similar extent than chelators. Hence, it is possible that the reduction of the oxidative tone by iron sequestration underlies one of the mechanisms by which chelators promote axodendritic rescue.

In addition to increased ROS levels, high intracellular calcium concentrations are intimately associated with the progress of neuronal degeneration observed in PD [48, 49]. Increasing iron levels in primary hippocampal neurons promotes calcium release from the endoplasmic reticulum [50], causing significant calcium dyshomeostasis and mitochondrial fragmentation [51], two factors that presumably contribute to the impairment of neuronal function produced by iron accumulation. Accordingly, iron chelators may also induce neurogenesis by decreasing toxic intracellular calcium levels to neuritogenesis-permissive concentrations.

In conclusion, this study provides evidence that treatment with iron chelators induces the regeneration of the axodendritic tree collapsed by MPP+ treatment, and restores nigrostriatal fibers and SNc cells in MPTP-treated mice. The design of chelator supplementation strategies leading to the regeneration of the axodendritic tree in damaged neurons may be relevant for the early treatment of PD, at stages of the disease where there is axodendritic tree retraction without neuronal death.

## Supporting Information

S1 Fig Synthesis strategy for 7DH and 7MH. ***Scheme 1*:** synthesis of 7DH. Reagents and conditions: (a) (CH_2_O)n, ethanol, 40°C, 10 min; (b) 8-hydroxyquinoline, reflux, 6 h. ***Scheme 2*:** synthesis of 7MH. Reagents and conditions: (a) (CH2O)n, morpholine, ethanol, 40°C, 10 min; (b) 8- hydroxyquinoline, reflux, 6 h. Purity of both 7DH and 7MH was <98% as determined by quantitative NMR.
(TIF)

S2 Fig Neurites restored by chelator treatment contain the presynaptic marker synaptophysin. Mesencephalic cells were treated for 24 h with 0.5 μM MPP+ followed by treatment for 48 h with 5 μM DFO, 50 μM DFP or 10 μM DPD. Cells were co-stained for TH (green) and synaptophysin (red). Nuclei labeling with TOPRO (blue) gives an account of the total cell population. The zoom columns depict enlargements of selected areas. Scale bar: 100 μm. The images are representative of similar findings displayed by four independent cultures.
(TIF)
