# Supplementary figures and images for: Iron Chelators and Antioxidants Regenerate Neuritic Tree and Nigrostriatal Fibers of MPP+/MPTP-Lesioned Dopaminergic Neurons
Source: PLoS One. 2015 Dec 14;10(12):e0144848. doi: 10.1371/journal.pone.0144848 (PMC4684383; doi:10.1371/journal.pone.0144848)

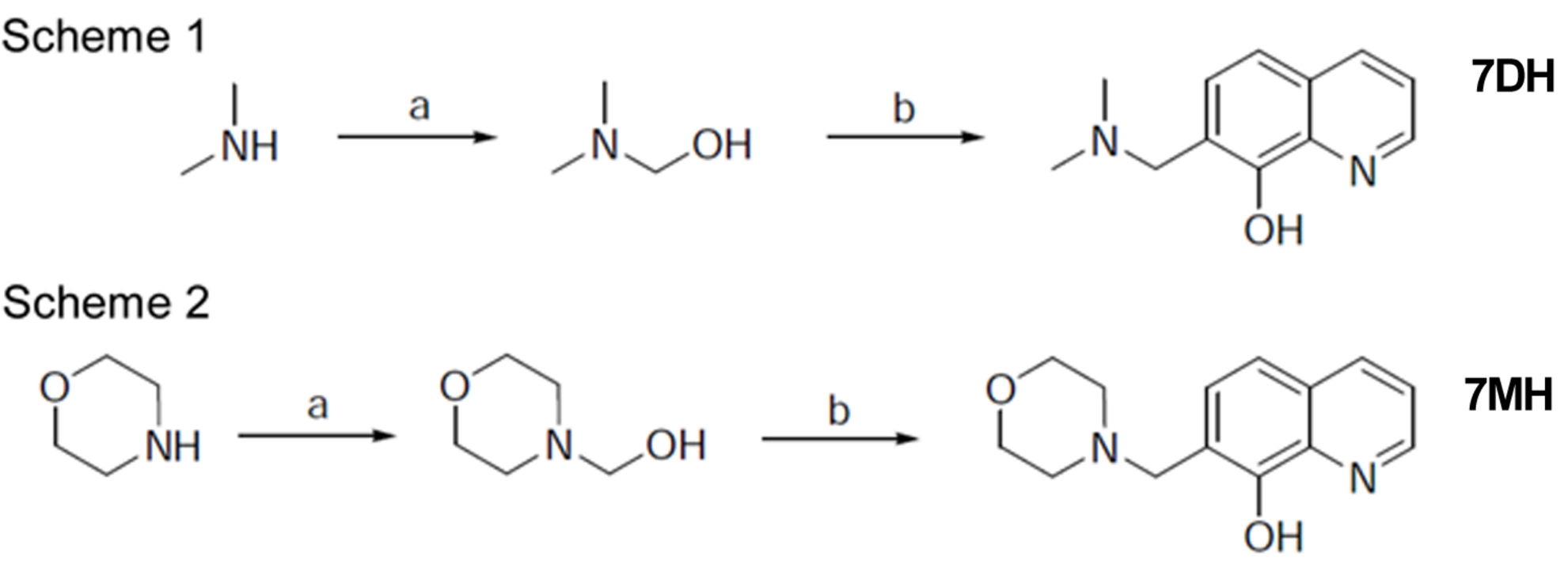

Supplement: S1 Fig — Scheme 1: synthesis of 7DH. Reagents and conditions: (a) (CH2O)n, ethanol, 40°C, 10 min; (b) 8-hydroxyquinoline, reflux, 6 h. Scheme 2: synthesis of 7MH. Reagents and conditions: (a) (CH2O)n, morpholine, ethanol, 40°C, 10 min; (b) 8- hydroxyquinoline, reflux, 6 h. Purity of both 7DH and 7MH was <98% as determined by quantitative NMR. (TIF) [file pone.0144848.s001.tif]

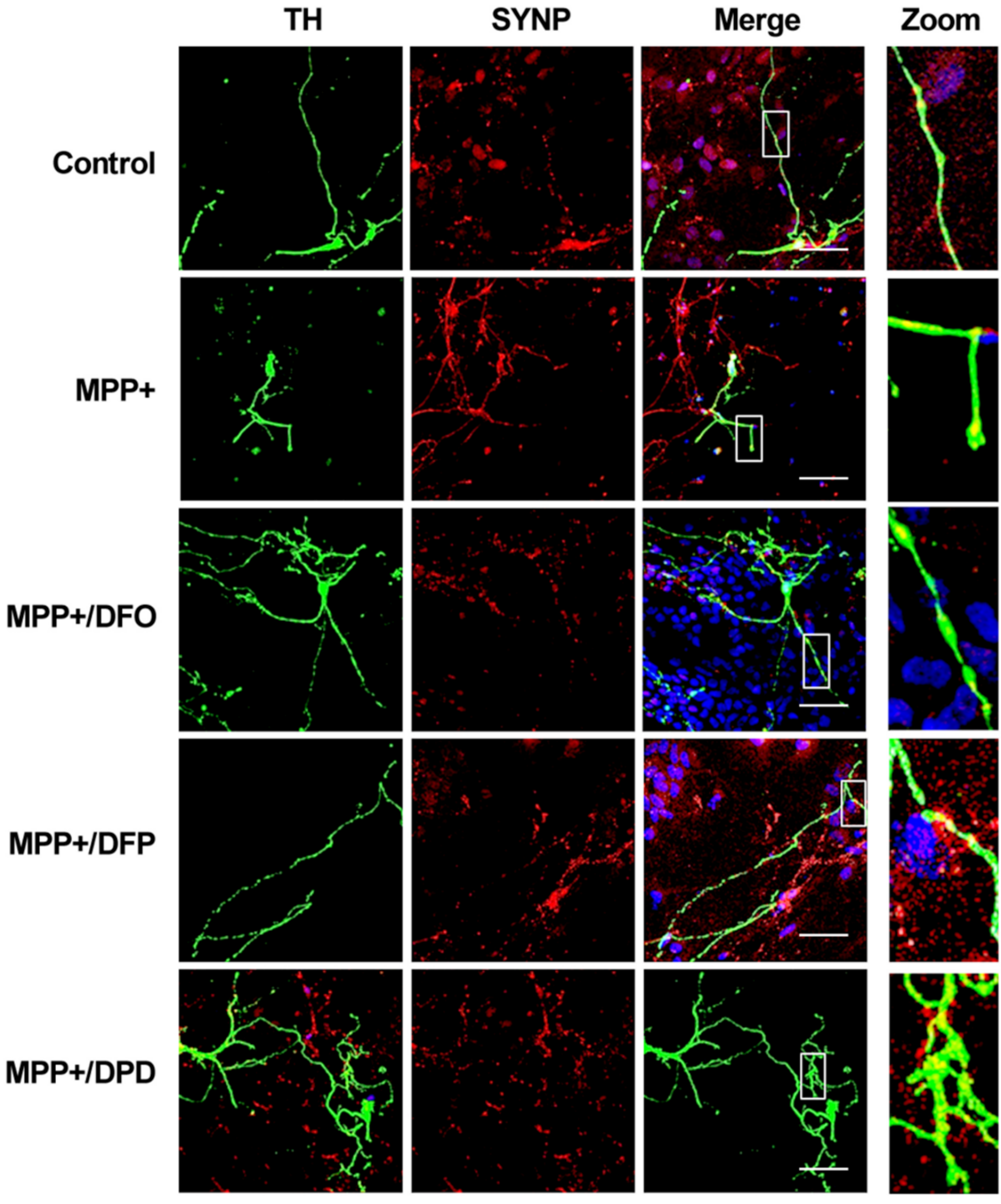

Supplement: S2 Fig — Mesencephalic cells were treated for 24 h with 0.5 μM MPP+ followed by treatment for 48 h with 5 μM DFO, 50 μM DFP or 10 μM DPD. Cells were co-stained for TH (green) and synaptophysin (red). Nuclei labeling with TOPRO (blue) gives an account of the total cell population. The zoom columns depict enlargements of selected areas. Scale bar: 100 μm. The images are representative of similar findings displayed by four independent cultures. (TIF) [file pone.0144848.s002.tif]
